# A Simplified Risk Assessment Tool to Predict Post Deceased Donor Liver Transplantation Outcomes: A Single, Highly Experienced Medical Center in Taiwan

**DOI:** 10.1002/kjm2.70136

**Published:** 2025-11-12

**Authors:** Jie‐Lan Jhang, Hao‐Chien Hung, Yin Lai, Jin‐Chiao Lee, Yu‐Chao Wang, Chih‐Hsien Cheng, Tsung‐Han Wu, Chen‐Fang Lee, Ting‐Jung Wu, Hong‐Shiue Chou, Kun‐Ming Chan, Wei‐Chen Lee

**Affiliations:** ^1^ School of Medicine, College of Medicine Chang Gung University Taoyuan Taiwan; ^2^ Division of Liver and Transplantation Surgery Chang‐Gung Memorial Hospital Taoyuan Taiwan

**Keywords:** deceased donor liver transplantation, donor risk index, overall survival, split liver transplantation

## Abstract

Liver transplantation (LT) is the standard treatment for end‐stage liver disease, yet the gap between the demand for organs and their availability is widening. In Taiwan, the scarcity of deceased donor organs highlights the need for optimized utilization strategies. The donor risk index (DRI) has emerged as a predictive tool for transplant outcomes, but existing models are not well‐suited for Taiwan's unique demographic and clinical context. This retrospective cohort study analyzed a total of 118 deceased donor liver transplantation (DDLT) cases from Chang Gung Memorial Hospital between January 2020 and October 2023. Key demographic and clinical data were collected, focusing on one‐year mortality and associated outcomes. Additionally, a retrospective validation cohort of 60 patients from January 2018 to December 2019 and a prospective validation cohort of 41 patients from November 2023 to August 2024 were included to assess the robustness of the CGMH‐DRI model. Statistical analyses included univariate and multivariate logistic regression to identify independent risk factors. The study identified MELD 3.0 score, donor total bilirubin, and cold ischemia time (CIT) as independent predictors of one‐year mortality. The CGMH‐DRI model demonstrated good predictive performance (AUC = 0.778) for mortality, early allograft dysfunction (EAD), and major complications. Validation results showed consistent performance, with AUROC values of 0.716 in the retrospective cohort and 0.694 in the prospective cohort for one‐year mortality; 0.781 and 0.676 for EAD; and 0.727 and 0.705 for major complications, respectively. The CGMH‐DRI model offers a simple yet helpful tool for risk stratification in DDLT. It enables clinicians to identify high‐risk patients and improve decision‐making in liver transplantation. Further validation in diverse populations is warranted to enhance its applicability.

## 1 Introduction

The standard treatment for patients with end‐stage liver disease, acute liver failure, and various stages of hepatocellular carcinoma is liver transplantation (LT) [1]. As LT is increasingly endorsed, the disparity between the number of people waiting for organs and their availability grows accordingly. The way that Eastern and Western nations approach the issue of scarcity varies. The need for transplants has considerably outpaced the supply of liver grafts, despite the commitment to raising the number of living donors. Despite an increase in the utilization of living donor liver transplantation (LDLT), there is still a need to optimize the use of organs from deceased donors. As a result, the split LT technique has been presented as the most effective manner to enable LT [2, 3], as splitting liver grafts from one single liver could safely benefit two adult recipients after accurate preoperative estimation [4].

Patient survival after receiving liver transplantation depends on many factors. As for deceased donor liver transplantation (DDLT), transplant expertise in the past was often faced with the difficult decision of whether to give this donor liver to the recipient. A robust assessment tool could provide more information to these physicians so that they can make wise decisions and adequately utilize the organ. Nonetheless, a minority overall of deceased donors of organs for LT exists in Asian nations, Taiwan included. Since the production of the donor risk index (DRI) [5] in 2006, there has been a trend toward establishing predictive models for liver transplantation a decade ago, such as the Eurotransplant Donor Risk Index (ET‐DRI) [6], the product of donor age and model for end‐stage liver disease (D‐MELD) [7], balance of risk (BAR) score [8], model for early allograft function (MEAF) [9], the liver graft assessment following transplantation (L‐GrAFT7) [10], the survival outcomes following liver transplantation (SOFT) score [11], and the improved donor‐to‐recipient allocation score for both deceased and living donors (ID^2^EAL‐DR) [12]. Most of the research focused on European and American countries. To continue, while highly informational, existing risk scoring systems with complex calculations are not easily translated into practical usage. Applying the models directly without considering different circumstances may contribute to less accuracy. There is no consensus yet on key donor and recipient factors for risk stratification in East Asia, including Taiwan. It highlights that developing a model for East Asia is an urgent task.

To address this problem, we are interested in discovering independent risks and developing a novel risk‐stratifying model, specifically for DDLT, to help predict short‐term outcomes in this era.

## 2 Materials and Methods

### 2.1 Study Population

This retrospective cohort analysis was carried out at the Linkou Chang Gung Memorial Hospital in Taiwan. From January 2020 to October 2023, a retrospective collection and review of consecutive liver transplant cases (*n* = 332) were conducted. We eventually enrolled 118 patients with DDLT after removing pediatric patients (*n* = 4), those who underwent living donor liver transplantation (LDLT; *n* = 209), and those who accepted donation after circulatory death (DCD; *n* = 1). In addition, a retrospective validation cohort of 60 patients from January 2018 to December 2019 and another prospective cohort of 41 patients from November 2023 to August 2024, tracked for at least 1 year, were analyzed to assess the CGMH‐DRI model's robustness and to confirm its utility in diverse clinical settings. Every transplant patient who is included follows up after surgery on a regular basis.

### 2.2 Data Extraction and Clinical Outcomes Measurement

The recipient's age, body mass index (BMI), sex, liver function, and etiology were among the basic demographics that were recorded. Other transplant factors were donor and graft details, graft‐to‐recipient weight ratio (GRWR), and surgical specifics. To ensure that the recipients' disease severity was presented accurately, the Child‐Pugh score, the model for end‐stage liver disease (MELD) score [13], the MELD‐sodium (MELD‐Na) score [14], and MELD 3.0 [15] were calculated based on the final preoperative information recorded. One‐year mortality following transplant was the main outcome that was evaluated. On the other hand, the following were the secondary outcomes: overall survival (OS) rates, length of stay (LOS), length of stay in the intensive care unit (ICU), rejection, severe surgical complications, 3‐month mortality, and early allograft dysfunction (EAD). Up to postoperative day (POD) seven, EAD‐associated variables such as aspartate transaminase (AST), alanine aminotransferase (ALT), serum total bilirubin, and international normalized ratio (INR) values were monitored at least once every day. Furthermore, in order to assess the discriminating power of our main result, data regarding three famous models were also collected: the donor age and recipient modified MELD (D‐MELD) score [16], DRI [5], and adjusted BAR score [8].

### 2.3 Definition of Associated Outcomes

Mortality is defined as all‐cause deaths rather than surgical‐associated ones. The definition of EAD is based on the one provided by Olthoff et al. [17]. It includes hyperbilirubinemia (> 10 mg/dL), extended INR (> 1.6) at POD7, and AST or ALT > 2000 IU/L over the first seven days. In our study, the use of pulse treatment during hospitalization—regardless of tissue confirmation through liver biopsy—was considered acute graft rejection. Postoperative problems were also documented in our study; major complications were classified as grades that matched or above the Clavien‐Dindo classification (CDC) [18] grade IIIb. To compare whether surgeons' overtime work influenced the post‐transplant outcome, the surgical start time was divided into ante meridiem (AM) and post meridiem (PM), with a cut‐off value of 12 o'clock at noon, assuming that the surgery finish time of the PM group would be beyond regular working hours. Cold ischemia time (CIT) and warm ischemia time (WIT) were recorded as the most common standard. The donor livers in our hospital were all preserved by static cold storage (SCS) method with histidine‐tryptophan‐ketoglutarate (HTK) solution [19].

### 2.4 Statistical Analysis

To present the data in categories and continuous forms, percentages (%) and mean values ± standard deviations with median, minimum to maximum levels were employed, respectively. The nonparametric method, the independent *T*‐test, and Pearson's chi‐square test were appropriately used to compare clinical parameters. The logistic regression analysis was utilized to investigate the effect of each parameter on mortality within a year following the transplant. The univariate (UV) analysis included all perioperative parameters; the multivariate (MV) analysis only included potential components with a *p*‐value < 0.100. A log‐rank test is used in conjunction with the Kaplan–Meier method to assess patient survival between groups. A receiver operating characteristic curve (ROC) was used to uncover the predictive value of a clinical component, and the Youden index was employed to ascertain the optimal cut‐off point for assessing predictive accuracy. All statistical analyses were performed using IBM SPSS version 24.0 (SPSS Incorporation, Chicago, IL, USA), and a two‐tailed *p*‐value of less than 0.05 was considered statistically significant.

## 3 Results

### 3.1 Characteristics of Enrolled Patients

This study comprised 118 transplants performed between 2020 and 2023. Table 1 displays the general data about the donors, recipients, and surgically related variables. With a mean BMI of 25.5 ± 5.6 kg/m^2^, the recipients' average age at transplantation was 50.6 ± 11.3 years, and 96 of them were male (79.7%). Viral hepatitis (*n* = 67, 56.8%) was the most common cause of liver illness, with alcohol‐related liver disease coming in second. Child‐Pugh class B (*n* = 53, 44.9%) and class C (*n* = 50, 42.4%) accounted for the majority of cases. The mean MELD‐Na score was 21.9 ± 9.8, the mean MELD 3.0 score was 22.5 ± 9.9, and the mean original MELD score was 21.0 ± 9.9 before LT. At the time of transplantation, 30 (25.4%) of the patients had HCC, 40 (33.9%) cases had prior abdominal surgery, and 16 (13.6%) recipients had portal vein thrombosis. About transplant donors, all liver donations were made after brain death (DBD), with the majority coming from our institution (*n* = 76, 64.4%). The mean age of donation was 41.9 ± 15.6 years, which was lower than that of recipients. Female donors constituted a smaller percentage (*n* = 42, 35.6%) than male donors. The donor BMI averaged 26.3 ± 5.4 kg/m^2^. Cerebrovascular accident (*n* = 76, 64.4%) was the most common cause of brain death at donation, with trauma coming in second (*n* = 16, 13.5%). The laboratory liver enzymes of the donor showed elevated mean AST of 115.5 ± 305.0 U/L and ALT of 96.7 ± 248.7 U/L with a mean total bilirubin of 1.0 ± 0.9 mg/dL, indicating that possible hepatic damage in various degrees is common for donors.

**TABLE 1:** Demographics of a total of 118 patients who underwent DDLT.

Variables	*M* ± SD, Mdn (min−max) or *n* (%)
Recipient factors	
Gender (male)	96 (79.7)
Age (years)	50.6 ± 11.3, 52 (18–66)
Body height (cm)	166.0 ± 9.9, 165.0 (122.0–186.0)
Body weight (kg)	70.8 ± 17.9, 70.0 (34.0–122.0)
BMI (kg/m^2^)	25.5 ± 5.6, 24.7 (15.6–44.8)
Primary liver disease	
Viral hepatitis	67 (56.8)
Alcoholic liver disease	33 (28.0)
Wilson's disease	2 (1.7)
Autoimmune hepatitis	1 (0.8)
Biliary atresia	3 (2.5)
Liver cancer	1 (0.8)
Others^a^	11 (9.3)
Child‐Pugh classification	
A/B/C	15 (12.7)/53 (44.9)/50 (42.4)
MELD score	
Original MELD	21.0 ± 9.9, 18 (6–40)
MELD‐Na	21.9 ± 9.8, 20 (6–40)
MELD 3.0	22.5 ± 9.9, 20.5 (6–40)
HCC	30 (25.4)
HBV infection	56 (47.5)
HCV infection	11 (9.3)
Previous abdominal surgery	40 (33.9)
PVT	16 (13.6)
Donor factors	
Sex (male)	94 (79.7)
Age (years)	41.9 ± 15.6, 43.5 (8–78)
Body height (cm)	167.6 ± 10.1, 167.0 (124.0–185.0)
Body weight (kg)	74.6 ± 18.2, 73.5 (26.0–115.0)
BMI (kg/m^2^)	26.3 ± 5.4, 26.0 (16.1–38.0)
CPCR	32 (27.1)
Cause of brain death	
Trauma	16 (13.5)
CVA	84 (71.2)
Anoxia	14 (11.9)
Others	4 (3.4)
Total bilirubin (mg/dL)	1.0 ± 0.9, 0.7 (0.3–6.4)
AST (U/L)	115.5 ± 305.0, 50 (7–3098)
ALT (U/L)	96.7 ± 248.7, 39 (4–2278)
BUN (mg/dL)	21.8 ± 16.1, 17.9 (5.0–121.8)
Creatinine (mg/dL)	1.7 ± 1.7, 1.2 (0.2–13.9)
Surgical factors	
Transplant type	
Whole liver	54 (45.8)
Split liver	64 (54.2)
GRWR (%)	
Left lobe	1.1 ± 0.3, 1.1 (0.6–1.9)
Right lobe	1.2 ± 0.2, 1.2 (0.7–1.6)
Ascites (mL)	3165.7 ± 485.3, 650 (0–23,800)
Allocation of graft origin	
Local (CGMH‐LK)	76 (64.4)
Region (North district of Taiwan)	32 (27.1)
National (Other districts of Taiwan)	10 (8.5)
Surgery start time^b^	
A.M.	87 (73.7)
P.M.	31 (26.3)
Operation time (h)	7.7 ± 1.9, 7.4 (3.8–15.8)
Cold ischemia time (h)	9.0 ± 5.0, 9.8 (1.2–23.6)
Warm ischemia time (h)	0.7 ± 0.2, 0.6 (0.4–1.6)
Spontaneous shunt ligation	9 (7.6)

Abbreviations: ALT, alanine aminotransferase; AST, aspartate aminotransferase; BMI, body mass index; BUN, blood urea nitrogen; CGMH‐LK, Chang Gung Memorial Hospital, Linkou branch; CVA, cerebrovascular accident; DDLT, deceased donor liver transplantation; GRWR, graft‐to‐recipient weight ratio; HBV, hepatitis B virus; HCC, hepatocellular carcinoma; HCV, hepatitis C virus; *M*, mean; max, maximum; Mdn, median; MELD, Model for End‐Stage Liver Disease; min, minimum; PVT, portal vein thrombosis; SD, standard deviation.

^a^ Idiopathic liver failure (*n* = 7), glycogen storage disease (*n* = 1), polycystic liver disease (*n* = 1).

^b^ AM stands for ante meridiem which is before midday. PM is for post meridiem which is after midday.

### 3.2 Post‐Transplant Outcomes and Risk Scores of Transplant Patients

The DRI, BAR, and D‐MELD scores as well as the postoperative outcomes for every transplant are presented in Table 2. The DRI, BAR, and D‐MELD scores had respective averages of 1.9 ± 9.9, 8.1 ± 5.2, and 872.7 ± 512.7. With an average ICU stay of 23.2 ± 74.2 days, the post‐transplant LOS was 37.5 ± 35.8 days on average. Thirty‐two (25.4%) of the cumulative fatality cases occurred within the first year following transplant, accounting for 32 cases (27.1%). Notably, more than half of the recipients infected with CMV during hospitalization (*n* = 68, 57.6%), nearly one‐third met the EAD criteria (*n* = 42, 35.6%), and approximately one‐quarter had major complications (*n* = 29, 24.6%). In our cohort, the acute rejection rate was 24.6% (*n* = 29).

**TABLE 2:** Calculations of the BAR, DRI, and D‐MELD scores with post‐transplant outcome.

Variables	*M* ± SD, Mdn (min‐max) or *n* (%)
Risk evaluation tools	
DRI score	1.9 ± 9.9, 1.95 (1.06–3.13)
BAR score	8.1 ± 5.2, 7.5 (1–19)
D‐MELD score	872.7 ± 512.7, 764.5 (117–2400)
Post‐transplant outcome	
OS (days)	534.4 ± 459.4, 396.5 (1–1556)
3‐month mortality	22 (18.6)
6‐month mortality	27 (22.9)
1‐year mortality	30 (25.4)
Died before last follow‐up date	32 (27.1)
ICU stay (days)	23.2 ± 74.2, 10.5 (1–142)
Length of stay (days)	37.5 ± 35.8, 26 (1–325)
EAD	42 (35.6)
Rejection	29 (24.6)
CMV during hospitalization	68 (57.6)
Complications^a^	
Any	49 (41.5)
Major (≥ grade IIIb)	29 (24.6)

Abbreviations: BAR, balance of risk; CMV, cytomegalovirus; D‐MELD, donor age × recipient Modified for End‐stage Liver Disease; DRI, donor risk index; EAD, early allograft dysfunction; M, mean; max, maximum; Mdn, median; min, minimum; OS, overall survival; SD, standard deviation.

^a^ Based on the Clavien‐Dindo classification (CDC) system of surgical complications.

### 3.3 Independent Risks Affecting Early Mortality After Transplant

**FIGURE 1:**
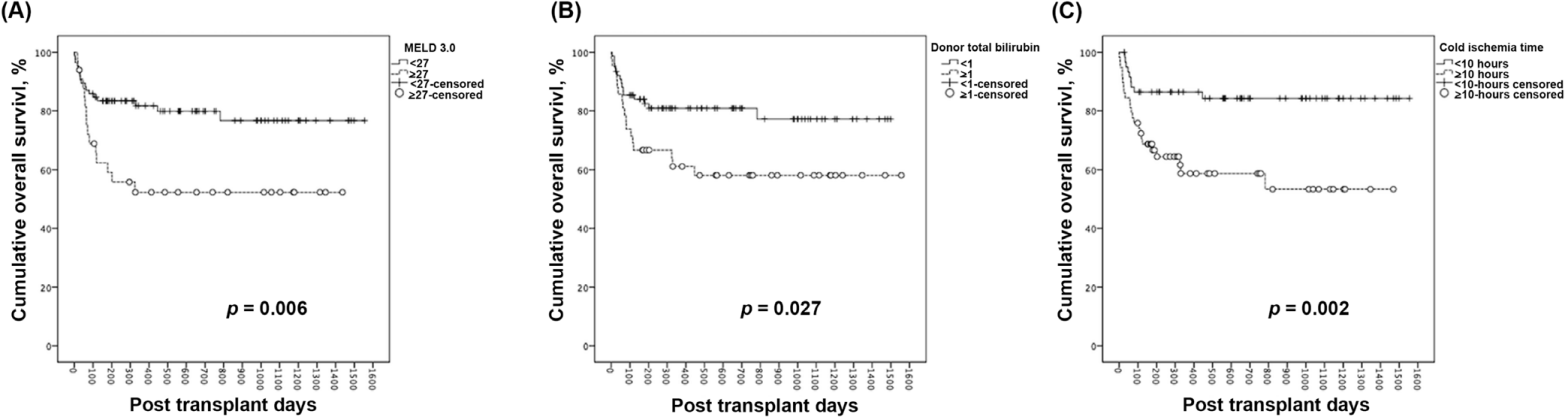
Kaplan–Meier plots of OS according to dichotomized subgroups, including (A) Recipient pre‐transplant MELD 3.0 score ≥ 27 and < 27, *p* = 0.006, (B) Donor serum total bilirubin ≥ 1.0 and < 1.0 mg/dL, *p* = 0.027, and (C) the CIT ≥ 10 and < 10 h, *p* = 0.002. CIT, cold ischemia time; MELD, model for end‐stage liver disease; OS, overall survival.

All collected pre‐transplant clinical parameters in Table 1 were subjected to univariate analysis, and those with potential significance (*p*‐value < 0.100) were entered into multivariate analysis to identify independent risks for one‐year mortality, as expressed in Table 3. In univariate analysis, of all the available clinical factors evaluated, five factors were significantly associated with the one‐year mortality after transplant. Five factors (including three versions of the MELD score with high repeatability) were found to be significantly associated with one‐year mortality after DDLT in the univariate analysis. These potential factors were then included in the multivariate analysis, which confirmed MELD 3.0 (OR = 5.294; 95% CI = 1.871–13.872; *p*‐value = 0.001), donor total bilirubin (OR = 3.127; 95% CI = 1.189–8.227; *p*‐value = 0.021), and CIT (OR = 3.979; 95% CI = 1.488–10.641; *p*‐value = 0.006) as independent risks for one‐year mortality. In Figure 1, the Kaplan–Meier plot of one‐year mortality from recipients with a MELD 3.0 score greater than 27, donors with a serum total bilirubin higher than 1.0 mg/dL, and allografts' CIT exceeding 10 h were significantly inferior (*p*‐value = 0.006, 0.027, and 0.003, respectively).

**TABLE 3:** Logistic regression for prediction of 1‐year mortality.

Variables (the optimal cut‐off value)	Univariate			
	*β* coefficient	OR	95% CI	*p*
Original MELD (20)	1.156	3.176	1.342–7.519	0.009
MELD‐Na (24)	1.137	3.118	1.326–7.333	0.009
MELD 3.0 (27)	1.358	3.889	1.607–9.428	0.003
Donor TB (1.0 mg/dL)	1.003	2.725	1.164–6.383	0.021
CIT (10 h)	1.379	3.972	1.593–9.928	0.003

Variables (the optimal cut‐off value)	Multivariate			
	*β* coefficient	OR	95% CI	*p*
MELD 3.0 (27)	1.628	5.294	1.871–13.872	0.001
Donor TB (1.0 mg/dL)	1.140	3.127	1.189–8.227	0.021
CIT (10 h)	1.381	3.979	1.488–10.641	0.006

*Note*: All preoperative parameters in Table 1 were calculated in univariate analysis, and only significant results (*p* < 0.100) are shown in this table and evaluated in multivariate analysis.

Abbreviations: CI, confidence interval; CIT, cold ischemia time; MELD, model for end‐stage liver disease; OR, odds ratio; TB, total bilirubin.

The three independent risk factors were further assessed using the performance indices presented in Table S1. The MELD 3.0 score demonstrated a sensitivity of 54.8%, a specificity of 77.4%, a positive predictive value (PPV) of 47.2%, and a negative predictive value (NPV) of 82.3% at an optimal cut‐off value of 27. For donor total bilirubin (mg/dL) and cold ischemic time (hours), the respective cut‐off values were 1 and 10. These parameters exhibited a sensitivity of 48.4% and 77.4%, specificity of 72.6% and 58.3%, PPV of 39.5% and 40.7%, and NPV of 79.2% and 87.5% in predicting one‐year mortality following DDLT.

### 3.4 Development of a CGMH‐DRI Risk Model and Its Predictive Value

**FIGURE 2:**
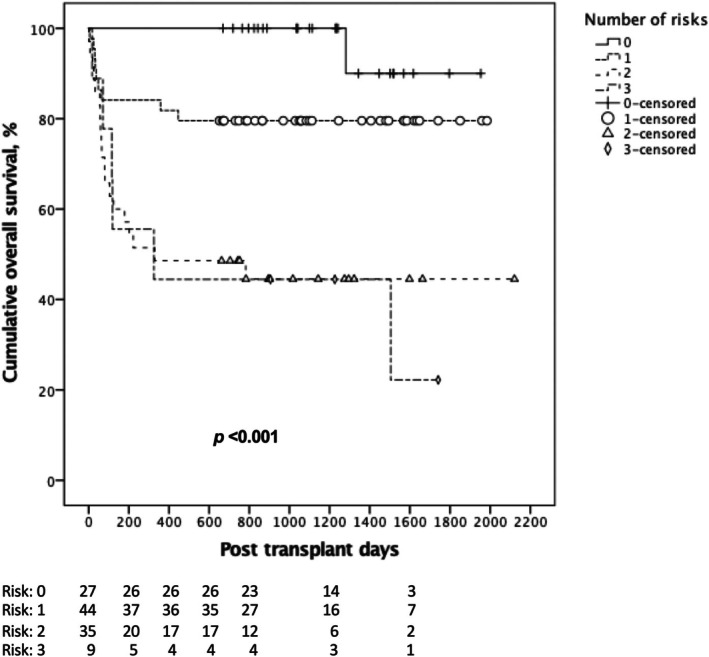
The significant impact of numbers of risk on OS. The curves are easy to divide into three groups visually (risk number = 0, 1, and ≥ 2). Significantly inferior survival outcomes were noted when the number was greater than or equal to 2, compared to the other two groups (*p* < 0.001).

**FIGURE 3:**
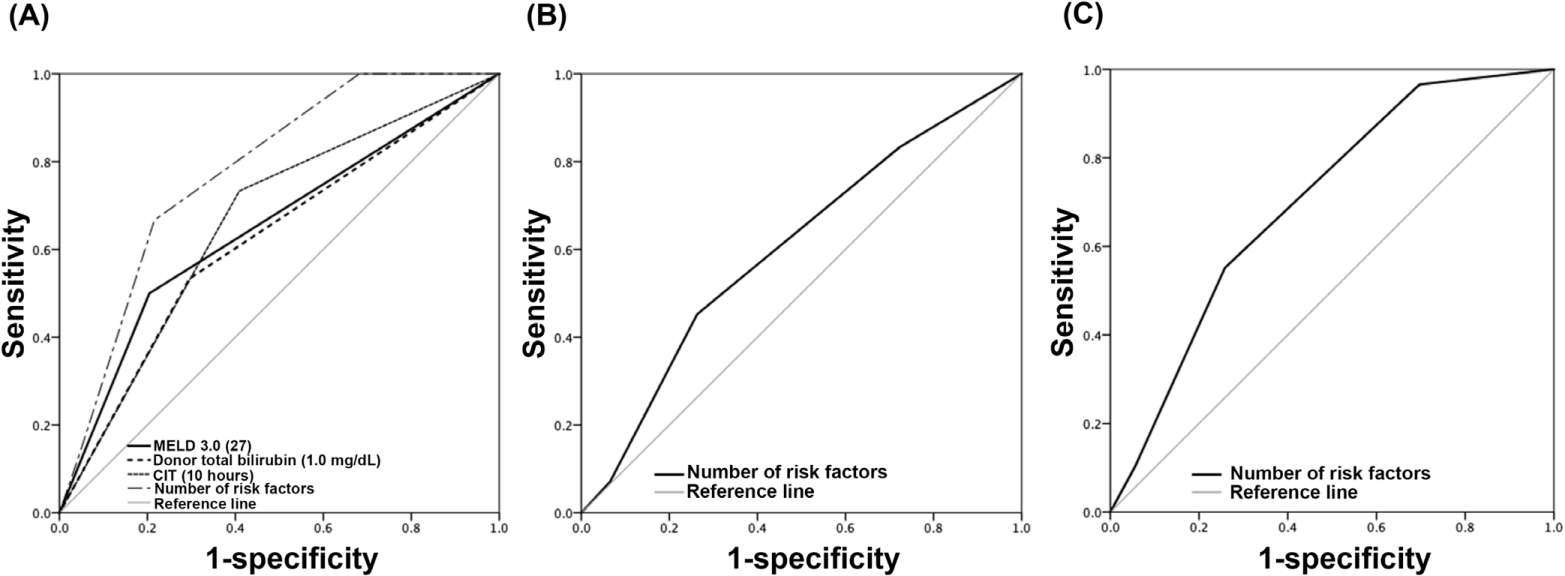
(A) The predictive value of the independent risks and number of containing risks (the CGMH‐DRI) in discriminating 1‐year mortality after DDLT. The AUROCs of individual risks were as follows: MELD 3.0, (AUC, [95% CI]) = 0.648 (0.528–0.768); Donor total bilirubin, (AUC, [95% CI]) = 0.619 (0.500–0.738); and CIT (AUC, [95% CI]) = 0.662 (0.551–0.773). Our proposed CGMH‐DRI model also showed great discriminative ability with an AUROC of 0.778 (95% CI, 0.690–0.865). Also, (B) an AUROC of 0.603 (95% CI: 0.497–0.710) and (C) an AUROC of 0.700 (95% CI: 0.598–0.801) correlate to EAD event and major surgical complication occurrence, respectively. AUROC, area under ROC; CI, confidence interval; CIT, cold ischemic time; DDLT, deceased donor liver transplantation; DRI, donor risk index; EAD, early allograft dysfunction; MELD, model of end liver disease; ROC, receiver operating characteristic.

After that, we developed a simplified CGMH‐DRI model for post‐transplant one‐year mortality by simply summing the risk number. In Figure 2, curves (risk number = 0, 1, and ≥ 2) can be visually separated into three categories with ease. According to the prognostic results, we define the risk index as: low risk (no risk factors), intermediate risk (including one risk factor) and high risk (including two or more risk factors). There was a significant decline in survival rate in the high‐risk group, while the low‐risk group demonstrated the best survival probability (*p* < 0.001). The ROC of our CGMH‐DRI model and its each contained risk is shown in Figure 3A. The AUROC of the model to predict one‐year mortality is 0.778 (95% CI: 0.690–0.865). Notably, the model also provides additional prognostication values in EAD (AUROC = 0.603; 95% CI = 0.497–0.710) and major complication event (AUROC = 0.700; 95% CI = 0.598–0.801), shown in Figure 3B,C.

### 3.5 Validation Cohorts Characteristics

We further established a retrospective validation cohort comprising 60 patients, alongside a prospective validation cohort of 41 patients, both of which were distinct from the derived group. As detailed in Table S2, nearly 70% of recipients in both cohorts were male, with mean ages of 52.8 ± 9.4 years for the retrospective cohort and 55.0 ± 9.0 years for the prospective cohort. Within these groups, 48.3% and 39.0% were categorized as Child‐Pugh Class C, respectively, while the average MELD scores were 20.5 ± 9.0 for the retrospective cohort and 18.9 ± 8.3 for the prospective cohort. Notably, 25 out of 60 patients in the retrospective cohort underwent split liver graft transplantation, compared to 24 out of 41 patients in the prospective cohort. The average CIT was recorded at 7.7 ± 3.3 h for both cohorts. Overall, a comparative analysis of patient demographics, surgical‐related data, and outcomes, as presented in Table 1, revealed no significant differences between the two validation groups and the derived group.

Utilizing the previously established CGMH‐DRI model—which incorporates identified risk factors such as MELD ≥ 27, donor serum total bilirubin > 1.0 mg/dL, and CIT > 10 h—we observed that among the 60 patients in the retrospective cohort, 20 did not exhibit any risk factors, 27 had one, 9 had two, and 4 presented with all three risk factors. In the prospective cohort of 41 patients, 15 did not exhibit any risk factors, 16 had one, 9 had two, and 1 presented with all three risk factors. Figure S1 illustrates the significant impact of various risk factors on OS in both validation groups. The graph distinctly delineates survival curves for different groups based on the number of risk factors (0, 1, 2, and 3). An increase in the number of risk factors was associated with notable declines in survival outcomes, with statistically significant differences observed (*p* = 0.002 for the retrospective cohort and *p* = 0.034 for the prospective cohort).

### 3.6 Validation of the CGMH‐DRI Model

Figures S2 and S3 illustrate the validation of the CGMH‐DRI model within both the retrospective and prospective cohorts, particularly focusing on patients with a high pre‐operative MELD score of ≥ 20. In terms of predicting (A) first‐year mortality, (B) EAD, and (C) major surgical complications following DDLT, the area under the receiver operating characteristic (AUROC) values were recorded as follows: 0.716 (95% CI: 0.547–0.884) for 1‐year mortality in the retrospective cohort and 0.694 (95% CI: 0.505–0.882) for the prospective cohort; 0.781 (95% CI: 0.650–0.912) for EAD events in the retrospective cohort and 0.676 (95% CI: 0.500–0.921) for the prospective cohort; and 0.727 (95% CI: 0.539–0.914) for major surgical complications in the retrospective cohort and 0.705 (95% CI: 0.489–0.921) for the prospective cohort. Notably, subgroup analyses indicated an improved capacity to distinguish between eventful and non‐eventful patients among those with a pre‐operative MELD score of ≥ 20. The AUROC values for (D) 1‐year mortality, (E) EAD events, and (F) major surgical complications in the retrospective group were 0.805 (95% CI: 0.593–1.000), 0.890 (95% CI: 0.753–1.000), and 0.909 (95% CI: 0.794–1.000), respectively. In the prospective group, these values were 0.889 (95% CI: 0.735–1.000), 0.827 (95% CI: 0.628–1.000), and 0.920 (95% CI: 0.791–1.000). The validation of the CGMH‐DRI model underscores its efficacy in predicting critical outcomes.

Figure S4 reveal calibration plots illustrating the differences between observed and predicted mortality events within 1 year after DDLT, which are presented for (A) the derived cohort, (B) the retrospectively validated cohort, and (C) the prospectively validated cohort. Furthermore, calibration plots for patients with a MELD score of ≥ 20 are shown for (D) the derived cohort, (E) the retrospectively validated cohort, and (F) the prospectively validated cohort. We evaluated the calibration performance of the predictive model using the Hosmer–Lemeshow goodness‐of‐fit test. The results indicate that for each diagram, the differences between model predictions and actual observations were not statistically significant, demonstrating a robust calibration capability of the predictive model.

## 4 Discussion

This single‐center analysis studied the short‐term outcomes of Taiwanese DDLTs accomplished over the near past years and aims to develop a CGMH‐DRI model to aid in future therapeutic decision‐making. Our institute is experienced at handling DDLT, especially the split ones, which have become a safe and helpful strategy for the shortage of liver grafts [20]. With the development of medical technology and the improvement of the intensive care unit care system, the prognosis of LT gradually improves. DRI should be adapted to local conditions and make inevitable adjustments in response to different national conditions. Taiwan is a unique place, with a closed island environment, convenient transportation, a high density of medical institutions, a comprehensive public health insurance system, a Chinese family structure, cultural traditions and folk beliefs, etc. To our knowledge, this study is Taiwan's first study regarding DRI in DDLT. As a result, our study also reflected a synergism of a multifactorial medical environment. A major character of the present study is to create the CGMH‐DRI model yielding good predictive performance (AUC = 0.778) without complicated mathematical processes by counting the number of risks. It is simple and convenient for clinicians to use. Similar predictive qualities were delivered [16].

The three independent risks identified are MELD 3.0, donor total bilirubin, and CIT. The original MELD score has long been used as a reliable indicator of short‐term survival in patients with end‐stage liver disease. MELD 3.0, as an iteration of the original MELD and MELD‐Na, reasonably provides a more accurate mortality prediction in general. Nevertheless, the correlation between original MELD, MELD‐Na, and MELD 3.0 should be clarified, though MELD 3.0 emphasizes that it has been rescaled to maintain the “MELD intuition” [15]. In the context of this study, we determined the best cut‐off values of 20 and 27 for the original MELD and MELD 3.0, respectively. It will be considered a high‐risk group if the score is higher than the decided value, of which the concept is also the foundation of the current study. We also found that EAD and worse SOFT are generally associated with increasing CITs [21, 22]. The cut‐off of previous research mainly lies in groups longer than 12 h, while our results demonstrated 10 h. Interestingly, CIT is not a definite known factor at the time of the organ acceptance decision but has a high hazard ratio among many studies. It is also an assembly of many elements in transplantation. For instance, fragile tissue, continuous bleeding during surgery, or obstacles during recipient surgery would eventually contribute to the final CIT. Bilirubin is one of the laboratory indices related to liver function. Livers obtained using extended criteria, such as donors with abnormal liver function tests, were associated with higher rates of graft loss. Yet, a previously published study revealed that no significant associations were found between total bilirubin, indirect bilirubin, and patients with nonalcoholic fatty liver disease [23].

In our study, we analyzed a total of 113 recipients, including 60 split liver procurement surgeries. Only one donation involved ex situ division of the liver parenchyma, with a bench surgery time of 3.60 h. The corresponding CITs for the grafts were 6.01 h for the right liver graft and 5.38 h for the left liver graft, both of which resulted in smooth recoveries. We found that cold ischemic time for split liver transplantation (9.20 ± 4.51 h) and whole liver transplantation (8.83 ± 5.53 h) did not show a significant difference (*p* = 0.701), and major complications were also similar between the two groups (*p* = 0.206). The role of split liver transplantation as a clinical factor influencing outcomes in DDLT remains uncertain. Several studies [24, 25] have similarly concluded that there is no significant difference in outcomes when comparing split liver transplantation to whole liver transplantation. Currently, the literature on the DRI in liver transplantation has limited references to split LT as a risk factor. However, a few studies [5] have explored the impact of split liver usage on recipient outcomes. Our derived cohort and our longitudinal cohort spanning 20 years also demonstrate no significant differences in outcomes. Our findings also suggest that the predominant use of in situ liver parenchyma division may contribute to the lack of significant differences observed between split and whole liver transplantation outcomes.

Several previously developed scoring models, such as DRI [5], BAR [8], and D‐MELD [7], score to predict SOFT [11], and improved donor‐to‐recipient allocation scores for both deceased and living donors (ID^2^EAL‐DR) [12], have shown validity in many studies published by different liver transplantation centers [26]. The identified relevant variables to the field of post‐transplant survival include re‐transplantation, recipient BMI, recipient medical condition in ICU, recipient liver etiology, recipient blood transfusion during hospitalization, recipient malignancy, recipient underlying disease, recipient PVT, donor age, donor height, donor weight, race, donor catecholamine index, donor maximum sodium level, donor cause of death, allocation of donor origin, split graft, and the era of transplant…etc. [16]. We assumed that the absence of correlation to patient survival in these variables in our research might be that we only included Taiwanese donors and recipients without other races (racial differences are limited), and selected cases in recent years rather than consecutive cases spanning more than 10 years (a concentrated transplant era with unified medical care background).

The validation of the CGMH‐DRI model provides insights into its capacity to predict key outcomes, including first‐year mortality, EAD, and major surgical complications following DDLT. The AUROC values suggest a modest level of performance for the model, particularly in identifying patients at high risk, especially those with elevated pre‐operative MELD scores. However, it is important to note that the predictive performance for EAD and major complications was modest, with AUROC values ranging from approximately 0.6 to 0.7. Importantly, further robust calibration performance also indicates the model's capability to align predicted outcomes with observed events. This reinforces the model's utility in clinical settings, as it can effectively stratify risk among patients. While the CGMH‐DRI model may serve as a valuable tool for clinicians in risk stratification and patient management in liver transplantation, further studies are necessary to enhance its applicability and refine risk assessment strategies across diverse patient populations. Continued validation in broader cohorts will help confirm its effectiveness and support its integration into clinical practice.

There were several limitations in this study due to its retrospective nature. In addition, the results were obtained from a single‐center experience. Machine perfusion for the graft is not a commonly used technology in our country and has not yet been included in our health insurance payment system. In the foreseeable future, the machine perfusion technique is definitely an important variable that needs to be taken into account and may be impactful on risk re‐stratification. In addition, this study cannot cover all models published in the past literature for comparison. To validate our findings, additional large‐scale, prospective, serial investigations involving other populations are required.

In conclusion, through a detailed analysis in response to current conditions, we propose a CGMH‐DRI model with great discriminatory power to predict one‐year mortality after DDLT. The risk‐stratified method is simple and predictive. It can help physicians by timely identifying patients at increased risk.

## Ethics Statement

The study was conducted according to the guidelines of the Declaration of Helsinki and approved by the Institutional Review Board of Chang‐Gung Memorial Hospital (IRB No. 202401417B0).

## Conflicts of Interest

The authors declare no conflicts of interest.

## Supporting information


**Figure S1:** This figure demonstrated how many risk factors have a major effect on OS after DDLT. Generally, as the risk number rose, a noticeably worse survival outcome was seen. The left panel (A) illustrates the results from the retrospective validation group, where survival analysis shows significant differences among varying risk numbers (0, 1, 2, 3) with a total *p*‐value of 0.002. The right panel (B) presents findings from the prospective validation group, revealing similar results, with significant differences in survival analysis across different risk numbers, yielding a total *p*‐value of 0.034. DDLT, deceased donor liver transplantation; OS, overall survival.

**Figure S2:** To validate the CGMH‐DRI model for the retrospective cohort in predicting (A) the first‐year mortality, (B) EAD, and (C) major surgical complications after DDLT. The AUROC values were 0.716 (95% CI: 0.547–0.884), 0.781 (95% CI: 0.650–0.912) and 0.727 (95% CI: 0.539–0.914) correlating to the 1‐year mortality, EAD event and major surgical complication occurrence, respectively. Interestingly, we found that the ability of distinguish between eventful and non‐eventful individuals seemed to increase in subgroup analysis. The AUROC values for (D) 1‐year mortality, (E) EAD event, and (F) major surgical complication occurrence for patients with a high pre‐operative MELD score ≥ 20 were 0.805 (95% CI: 0.593–1.000), 0.890 (95% CI: 0.753–1.000), and 0.909 (95% CI: 0.794–1.000), respectively. AUROC, area under ROC; CI, confidence interval; DDLT, deceased donor liver transplantation; DRI, donor risk index; EAD, early allograft dysfunction; MELD, model of end liver disease; ROC, receiver operating characteristic.

**Figure S3:** This figure validates the CGMH‐DRI model in a prospective cohort for predicting (A) first‐year mortality, (B) EAD, and (C) major surgical complications following DDLT. The area under the receiver operating characteristic (AUROC) values were 0.694 (95% CI: 0.505–0.882) for 1‐year mortality, 0.676 (95% CI: 0.500–0.921) for EAD events, and 0.705 (95% CI: 0.489–0.921) for the occurrence of major surgical complications. Notably, subgroup analysis revealed an enhanced ability to differentiate between individuals with and without events. For patients with a high pre‐operative MELD score of ≥ 20, the AUROC values for (D) 1‐year mortality, (E) EAD events, and (F) major surgical complications were 0.889 (95% CI: 0.735–1.000), 0.827 (95% CI: 0.628–1.000), and 0.920 (95% CI: 0.791–1.000), respectively. AUROC, area under ROC; CI, confidence interval; DDLT, deceased donor liver transplantation; DRI, donor risk index; EAD, early allograft dysfunction; MELD, model of end‐stage liver disease; ROC, receiver operating characteristic.

**Figure S4:** Simplified calibration plots visualizing the relationship between risk numbers (x‐axis) and the corresponding observed and predicted mortality cases within one‐year post‐Donation after Cardiac Death (DDLT) (y‐axis). The nearly overlapping lines representing predicted events based on risk numbers and actual observed events indicate the reliability of the calibration diagrams and the adequacy of the model fit. The upper three plots illustrate calibration for (A) the derived cohort, (B) the retrospectively validated cohort, and (C) the prospectively validated cohort. The lower three plots depict (D) the derived cohort, (E) the retrospectively validated cohort, and (F) the prospectively validated cohort, specifically focusing on patients with a Model for End‐Stage Liver Disease (MELD) score of ≥ 20.

**Table S1:** Accuracy analysis of clinical parameters in predicting one‐year mortality after liver transplantation.
**Table S2:** Demographics of the retrospective (*n* = 60) and prospective validation groups.

## Data Availability

The data presented in this study is available on request from the corresponding author.
