# Supplementary figures and images for: A Simplified Risk Assessment Tool to Predict Post Deceased Donor Liver Transplantation Outcomes: A Single, Highly Experienced Medical Center in Taiwan
Source: Kaohsiung J Med Sci. 2025 Nov 12;42(5):e70136. doi: 10.1002/kjm2.70136 (PMC13182605; doi:10.1002/kjm2.70136)

(A)

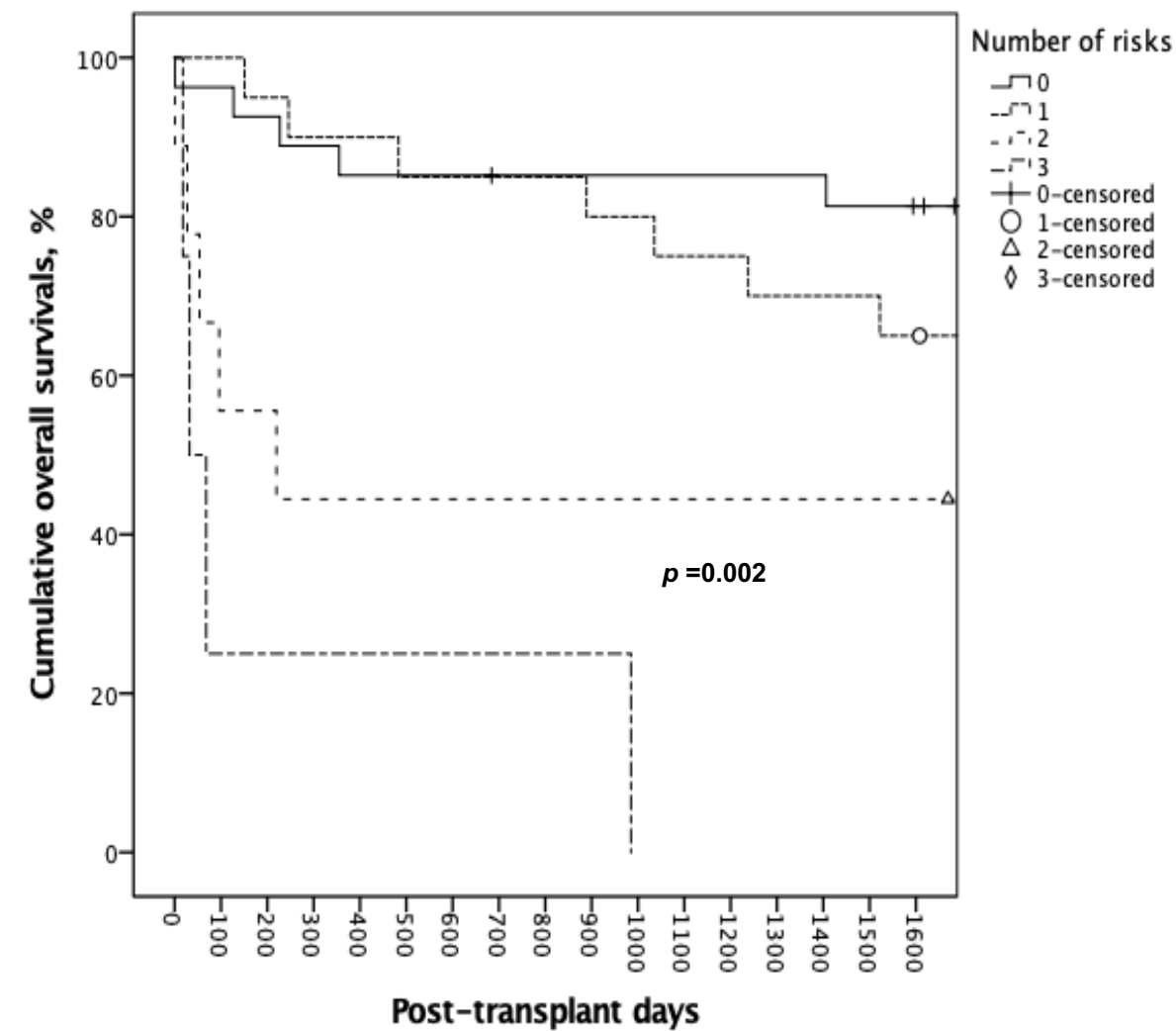

(B)

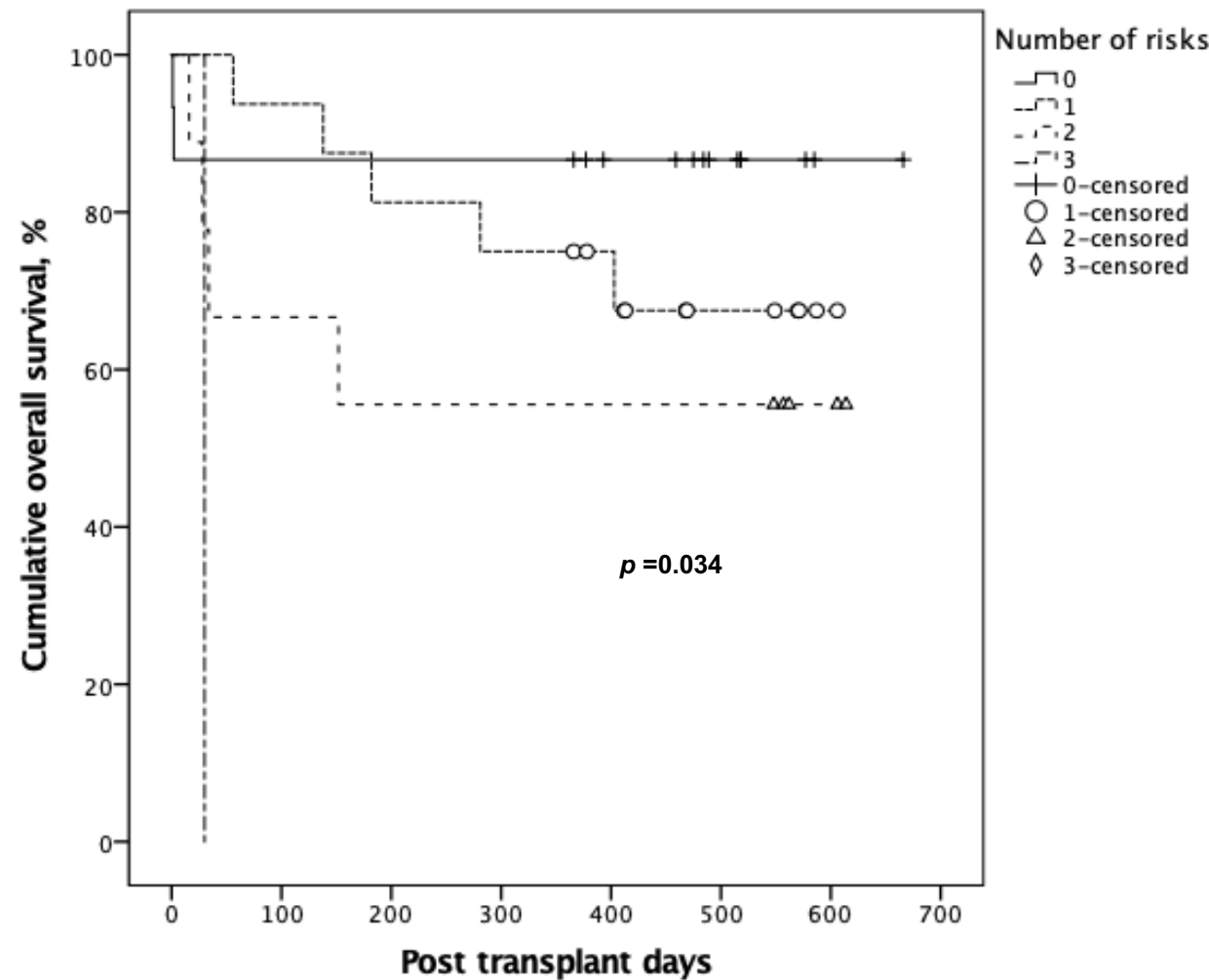

Supplement: Supplementary file 1 — Figure S1: This figure demonstrated how many risk factors have a major effect on OS after DDLT. Generally, as the risk number rose, a noticeably worse survival outcome was seen. The left panel (A) illustrates the results from the retrospective validation group, where survival analysis shows significant differences among varying risk numbers (0, 1, 2, 3) with a total p‐value of 0.002. The right panel (B) presents findings from the prospective validation group, revealing similar results, with significant differences in survival analysis across different risk numbers, yielding a total p‐value of 0.034. DDLT, deceased donor liver transplantation; OS, overall survival. [file KJM2-42-e70136-s003.pdf]

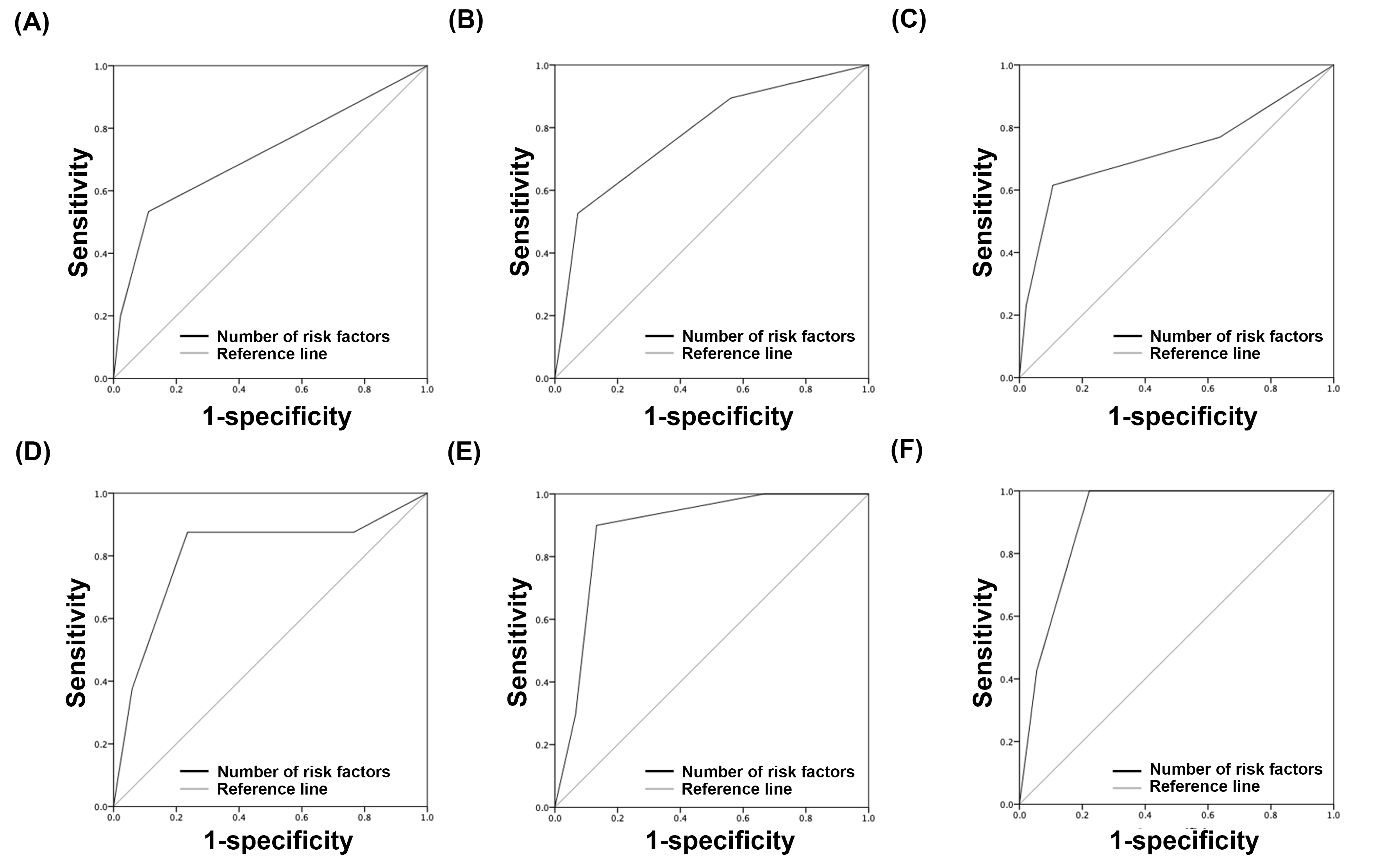

Supplement: Supplementary file 2 — Figure S2: To validate the CGMH‐DRI model for the retrospective cohort in predicting (A) the first‐year mortality, (B) EAD, and (C) major surgical complications after DDLT. The AUROC values were 0.716 (95% CI: 0.547–0.884), 0.781 (95% CI: 0.650–0.912) and 0.727 (95% CI: 0.539–0.914) correlating to the 1‐year mortality, EAD event and major surgical complication occurrence, respectively. Interestingly, we found that the ability of distinguish between eventful and non‐eventful individuals seemed to increase in subgroup analysis. The AUROC values for (D) 1‐year mortality, (E) EAD event, and (F) major surgical complication occurrence for patients with a high pre‐operative MELD score ≥ 20 were 0.805 (95% CI: 0.593–1.000), 0.890 (95% CI: 0.753–1.000), and 0.909 (95% CI: 0.794–1.000), respectively. AUROC, area under ROC; CI, confidence interval; DDLT, deceased donor liver transplantation; DRI, donor risk index; EAD, early allograft dysfunction; MELD, model of end liver disease; ROC, receiver operating characteristic. [file KJM2-42-e70136-s005.tif]

(A)

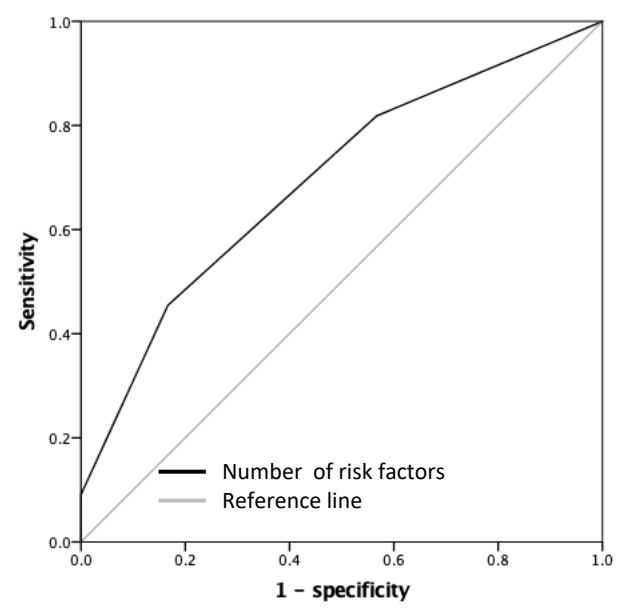

(B)

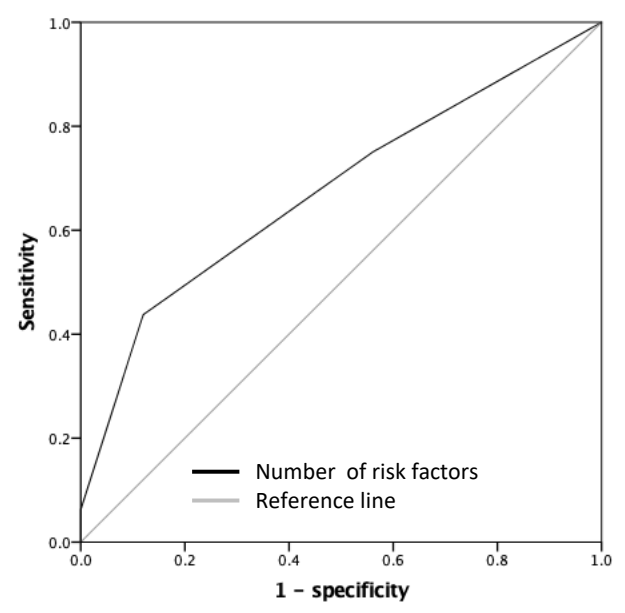

(C)

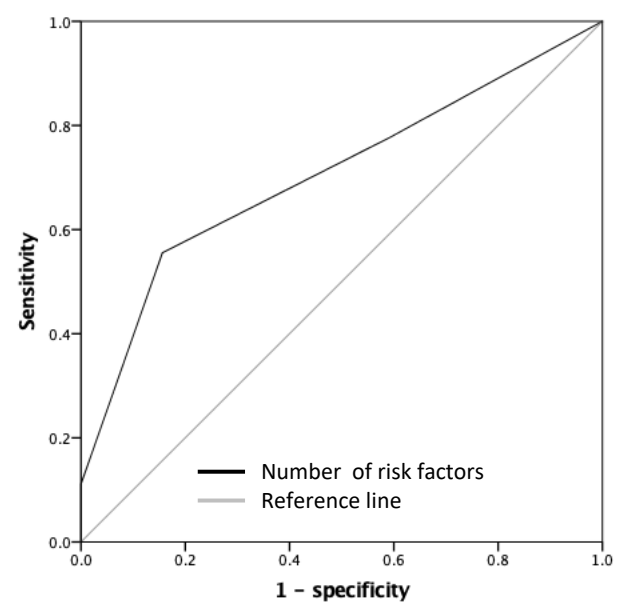

(D)

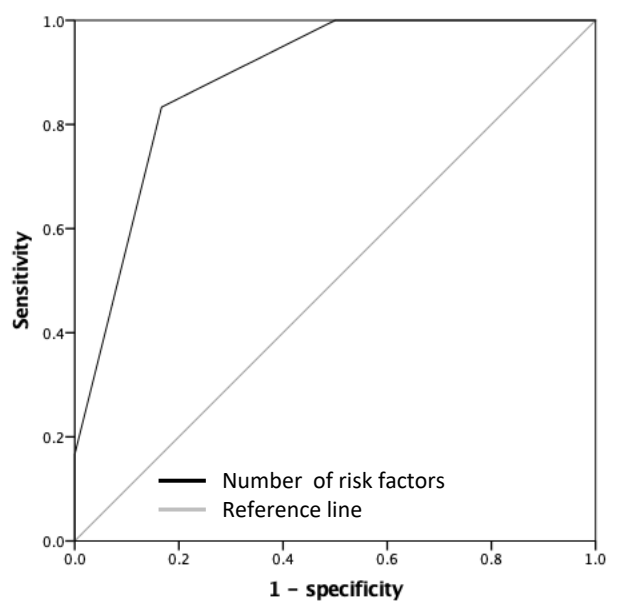

(E)

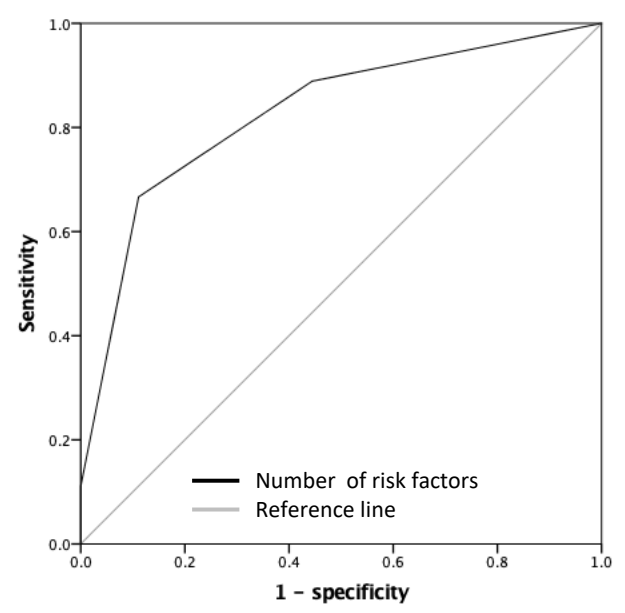

(F)

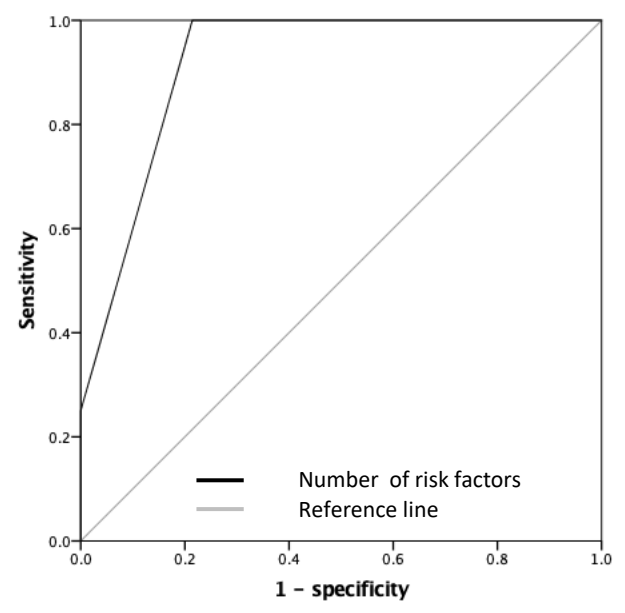

Supplement: Supplementary file 3 — Figure S3: This figure validates the CGMH‐DRI model in a prospective cohort for predicting (A) first‐year mortality, (B) EAD, and (C) major surgical complications following DDLT. The area under the receiver operating characteristic (AUROC) values were 0.694 (95% CI: 0.505–0.882) for 1‐year mortality, 0.676 (95% CI: 0.500–0.921) for EAD events, and 0.705 (95% CI: 0.489–0.921) for the occurrence of major surgical complications. Notably, subgroup analysis revealed an enhanced ability to differentiate between individuals with and without events. For patients with a high pre‐operative MELD score of ≥ 20, the AUROC values for (D) 1‐year mortality, (E) EAD events, and (F) major surgical complications were 0.889 (95% CI: 0.735–1.000), 0.827 (95% CI: 0.628–1.000), and 0.920 (95% CI: 0.791–1.000), respectively. AUROC, area under ROC; CI, confidence interval; DDLT, deceased donor liver transplantation; DRI, donor risk index; EAD, early allograft dysfunction; MELD, model of end‐stage liver disease; ROC, receiver operating characteristic. [file KJM2-42-e70136-s001.pdf]

**(A)**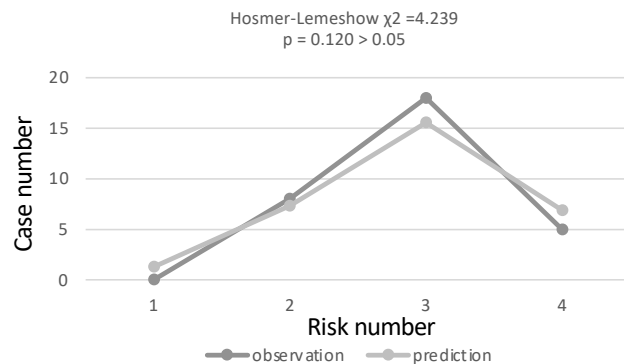**(B)**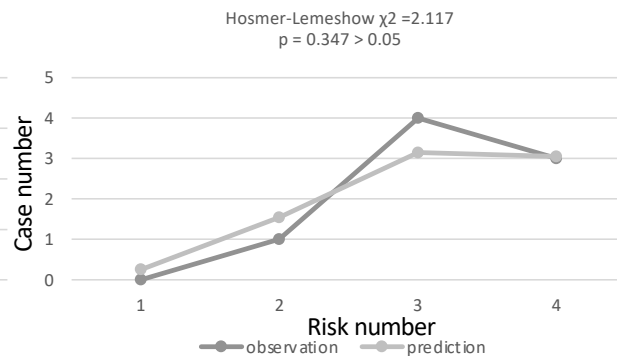**(C)**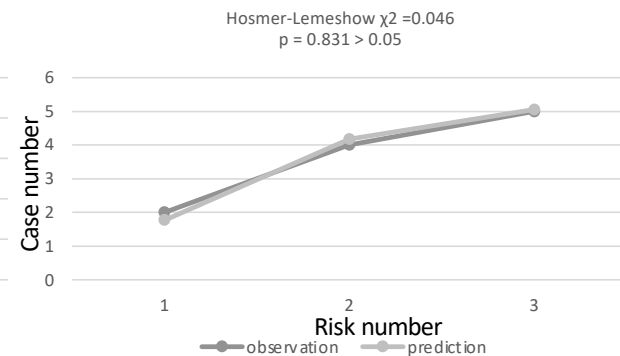**(D)**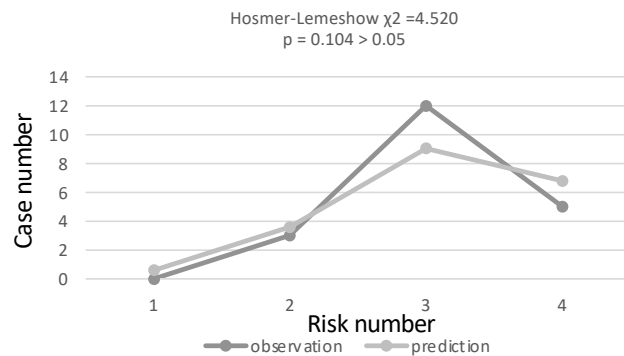**(E)**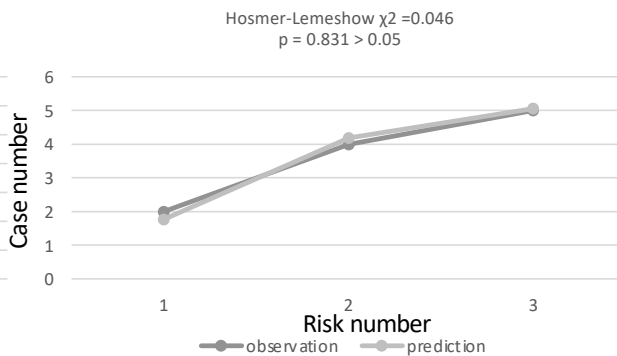**(F)**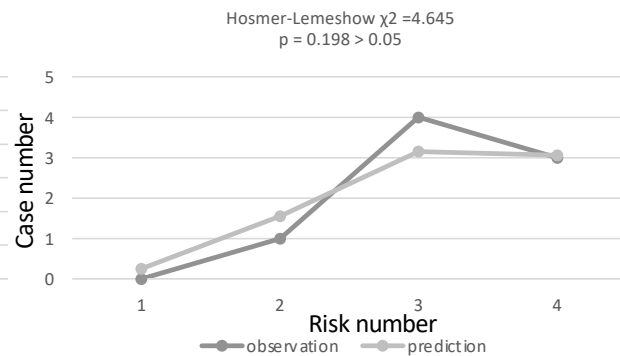

Supplement: Supplementary file 4 — Figure S4: Simplified calibration plots visualizing the relationship between risk numbers (x‐axis) and the corresponding observed and predicted mortality cases within one‐year post‐Donation after Cardiac Death (DDLT) (y‐axis). The nearly overlapping lines representing predicted events based on risk numbers and actual observed events indicate the reliability of the calibration diagrams and the adequacy of the model fit. The upper three plots illustrate calibration for (A) the derived cohort, (B) the retrospectively validated cohort, and (C) the prospectively validated cohort. The lower three plots depict (D) the derived cohort, (E) the retrospectively validated cohort, and (F) the prospectively validated cohort, specifically focusing on patients with a Model for End‐Stage Liver Disease (MELD) score of ≥ 20. [file KJM2-42-e70136-s002.pdf]
