# Supplementary material for: A Simplified Risk Assessment Tool to Predict Post Deceased Donor Liver Transplantation Outcomes: A Single, Highly Experienced Medical Center in Taiwan
Source: Kaohsiung J Med Sci. 2025 Nov 12;42(5):e70136. doi: 10.1002/kjm2.70136 (PMC13182605; doi:10.1002/kjm2.70136)
Supplement: Supplementary file 5 — Table S1: Accuracy analysis of clinical parameters in predicting one‐year mortality after liver transplantation. Table S2: Demographics of the retrospective (n = 60) and prospective validation groups. [file KJM2-42-e70136-s004.docx]

| **Supplemental table 1.** Accuracy analysis of clinical parameters in predicting one-year mortality after liver transplantation | | | | | |
| --- | --- | --- | --- | --- | --- |
| Parameters  (cut-off values) | AUC (95% CI); p values | Sensitivity | Specificity | PPV | NPV |
| MELD 3.0 (27) | 0.661 (0.544-0.778); p=0.008 | 54.8% | 77.4% | 47.2% | 82.3% |
| Donor TB (1.0) | 0.605 (0.485-0.725); p=0.085 | 48.4% | 72.6% | 39.5% | 79.2% |
| CIT (10) | 0.679 (0.571-0.786); p=0.003 | 77.4% | 58.3% | 40.7% | 87.5% |

**Abbreviation**: AUC = area under curve; CI = confidence interval; PPV = positive predictive value; NPV = negative predictive value; MELD = model of end liver disease; TB = total bilirubin, mg/dL; CIT = cold ischemic time, hours.

| **Supplemental Table 2.** Demographics of the retrospective (n = 60) and prospective validation groups | | |
| --- | --- | --- |
| Variables | M ± SD, Mdn (min-max) or n (%) | |
| Recipient factors | Retrospective validation  (n=60) | Prospective validation  (n=41) |
| Gender (male) | 41 (68.3) | 30 (73.2) |
| Age (years) | 52.8 ± 9.4, 55 (31-69) | 55.0 ± 9.0, 55 (37-67) |
| Child-Pugh classification |  |  |
| A/B/C  MELD score | 11/20/29 (18.3/33.3/48.3) | 10/15/16 (24.4/36.6/39.0) |
| Original MELD | 20.5 ± 9.0, 18 (8-40) | 18.9 ± 8.3, 18 (8-40) |
| MELD-Na | 21.2 ± 9.1, 19 (8-40) | 19.9 ± 9.1, 19 (8-40) |
| MELD 3.0 | 21.6 ± 9.3, 19 (8-40) | 19.9 ± 9.0, 19 (8-40) |
| Donor and surgical factors |  |  |
| Gender (male) | 45 (75.0) | 45 (75.0) |
| Age (years) | 41.8 ± 15.3, 43 (15-75) | 41.8 ± 15.3, 43 (15-75) |
| Total bilirubin (*mg*/*dL*) | 1.0 ± 0.9, 0.7 (0.1-3.9) | 1.0 ± 0.9, 0.7 (0.1-3.9) |
| Cold ischemia time (hours) | 7.7 ± 3.3, 7.9 (1.5-15) | 7.7 ± 3.3, 7.9 (1.5-15) |
| Transplant type |  |  |
| Whole liver/ Split liver | 35/25 (58.3/41.7) | 17/24 (41.5/58.5) |
| Post-transplant outcomes | | |
| EAD | 19 (31.7) | 16 (39.0) |
| Rejection | 18 (30.0) | 10 (24.3) |
| 1-year mortality | 15 (25.0) | 11 (26.8) |
| Died before last follow-up date | 21 (35.0) | 12 (29.3) |
| ICU stay (days) | 18.1 ± 8.9, 12 (1-96) | 16.5 ± 10.0, 12 (1-45) |
| Complications† |  |  |
| Any | 29 (48.3) | 21 (51.2) |
| Major (≥grade IIIb) | 13 (21.6) | 9 (22.0) |
| Risk evaluation (the cut-off value) | | |
| MELD 3.0 (27) | 17 (28.3) | 9 (22.0) |
| Donor TB (1.0 mg/dL) | 16 (26.7) | 14 (34.1) |
| CIT (10 hrs) | 25 (41.7) | 13 (31.7) |
| Number of risk factors  0/1/2/3 | 20/27/9/4  (33.3/45.0/15.0/6.7) | 15/16/9/1  (36.6/39.0/22.0/2.4) |
| **Abbreviation:** M = mean; SD = standard deviation; Mdn = median; min = minimum; max = maximum; MELD = Model for End-stage Liver Disease; OS = overall survival; EAD = early allograft dysfunction; TB = total bilirubin; CIT = cold ischemic time. | | |
| † Based on the Clavien-Dindo classification (CDC) system of surgical complications | | |
